# Combination of High- and Low-Rate GPS Receivers for Monitoring Wind-Induced Response of Tall Buildings

**DOI:** 10.3390/s18124100

**Published:** 2018-11-23

**Authors:** Zhonghai Yi, Cuilin Kuang, Yarong Wang, Wenkun Yu, Changsheng Cai, Wujiao Dai

**Affiliations:** 1 School of Geosciences and Info-Physics, Central South University, Changsha 410083, China; yizhonghai@163.com (Z.Y.); cscai@hotmail.com (C.C.); wjdai@csu.edu.cn (W.D.); 2 School of Geodesy and Geomatics, Wuhan University, Wuhan 430079, China; CSUwyr@163.com; 3 Department of Land Surveying and Geo-Informatics, The Hong Kong Polytechnic University, Hong Kong 310058, China; wk.yu@connect.polyu.hk

**Keywords:** high-rate GPS, wind-induced vibrations, time-differenced positioning, single-epoch relative positioning, precise point positioning

## Abstract

High-rise buildings are susceptible to wind-induced displacements, which can be precisely monitored by using GPS technology. However, GPS monitoring applications may be subject to signal interference and high hardware costs. This study presents a new wind-induced vibration monitoring approach that is based on the mixed use of high-rate and low-rate GPS receivers. In the proposed approach, high-rate receivers are only required in the monitoring stations, where we apply time-differenced positioning to obtain position changes between adjacent epochs. The derived high-rate monitoring station position changes are then integrated with low-rate single epoch relative positioning results between the monitoring and reference stations. Experimental results with both simulated and real data show that the proposed method has a comparable performance with the traditional relative positioning approach, in terms of determining buildings’ vibration frequency, displacement, and acceleration.

## 1. Introduction

Under influences such as operating load, strong wind, and earthquake, high-rise buildings normally experience vibrations and quasi-static deformations [1,2,3,4]. Continuous monitoring of the deformations is critical for the prevention of structure accidents and assessment of building design. Among the external loads, tall buildings are more susceptible to the wind. At the design stage, dynamic response tests are often carried out in the wind tunnel laboratory by means of simulation [5,6]. However, the test results often have large errors due to the difference between the laboratory and real world. Therefore, it is very important to measure the in-situ wind-induced vibrations of the high-rise buildings. For monitoring vibrations of high-rise buildings, there are some traditional sensors widely deployed, including accelerometer, laser collimator, tiltmeter, robotic total station, displacement transducer, and laser interferometer [7,8,9,10]. Compared with the traditional monitoring technologies, GPS has advantages such as high positioning accuracy, use in all-weather, no need for inter-visibility, and can directly offer the vibration displacement of buildings. In recent years, due to the rapid development of GPS hardware and software, GPS-based structural health monitoring has become a hot research topic in the fields of surveying and civil engineering.

At present, there are three major GPS positioning methods for monitoring the wind-induced responses of high-rise buildings, namely, single-epoch relative positioning method [10,11], real-time kinematic (RTK) method [12,13,14,15,16,17,18], and Precise Point Positioning (PPP) method [19,20,21,22,23]. The single-epoch relative positioning method effectively eliminates spatial-correlated errors thus can provide high-accuracy positioning results. However, this method requires high-performance and high-rate GPS receivers at both the monitoring and reference stations, which will increase the cost of engineering application. Furthermore, in an urban canyon, GPS satellite signals are usually disturbed by strong multipath errors and signal shadowing. Setting up a reference station near the monitoring sites on the buildings sometimes can be difficult. The RTK technique requires a data communication chain and the essence of RTK method is real time single-epoch relative positioning method. To overcome the shortcomings in single-epoch relative positioning method and RTK method, researchers propose to monitor wind-induced deformation of high-rise buildings by PPP method [19,20,21,22,23]. PPP can save the hardware cost without setting up the reference station. However, because PPP corrects GPS errors using model corrections or parameter estimation, its positioning accuracy is usually poorer than single-epoch relative positioning method and RTK method. As a result, the precise quasi-static deformation of the buildings cannot be accurately obtained [19,20,21,22,23].

We propose to combine high- and low-rate GPS receivers to address the above issues. In the new method, a high-rate GPS receiver is set up on the building roof to collect the high-rate observation data. A low-rate GPS receiver is used in the reference station. Generally, an urban Continuously Operating Reference System (CORS) station can meet this requirement, which not only solves the problem of selecting the reference station, but also saves the cost of the monitoring project. The coordinates of the monitoring station are computed by the high- and low-rate hybrid GPS positioning method. According to the difference of the monitoring station coordinates and its reference coordinate, the vibration displacement and the quasi-static component displacement of the tall building caused by the wind load can be calculated.

In the following sections, Section 2 presents the model of mixed use of high- and low-rate receivers. Section 3 and Section 4 show the results of simulation and real experiments, respectively. Section 5 gives some concluding remarks.

## 2. Combination of High- and Low-Rate GPS Receivers

**Figure 1:**
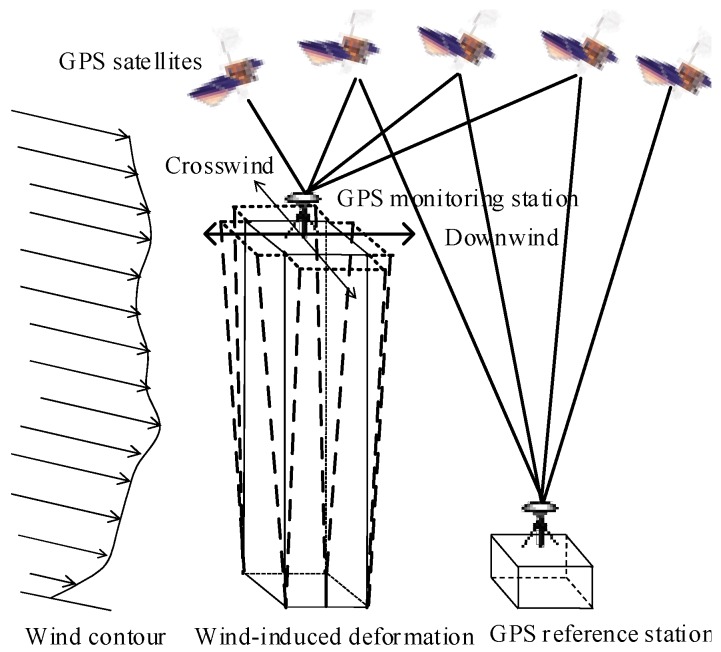
Scheme of monitoring wind-induced response of a tall building using GPS.

Figure 1 shows the scheme of monitoring wind-induced response of a tall-building using GPS. Under the action of wind load, high-rise buildings will experience deformations in the downwind direction and crosswind direction. The asymmetry of the building structure may cause bending and torsions. In general, a monitoring system consists of two GPS receivers, one is on the top of the building as a monitoring station, the other one is a reference station set upon a lower and more stable building. The distance between the two GPS stations should be as short as possible to more effectively reduce spatial-correlated errors. The dynamic deformation of the building is obtained in the single-epoch relative positioning where the reference position is usually measured by long-term GPS static positioning under negligible external wind loads. If both the reference station and monitoring station use a high-rate GPS receiver, it becomes the single-epoch relative positioning [10,11]. If the reference station and monitoring station constitute an RTK system, it is the RTK positioning [12,13,14,15,16,17,18]. If there are only GPS monitoring stations and no reference stations, it is the PPP positioning [19,20,21,22,23]. If the monitoring station uses a high-rate GPS receiver and the low-rate GPS receiver (usually a CORS station) is used as reference station, it is the monitoring wind-induced response method proposed in this paper by combining high- and low-rate GPS receivers. 

**Figure 2:**
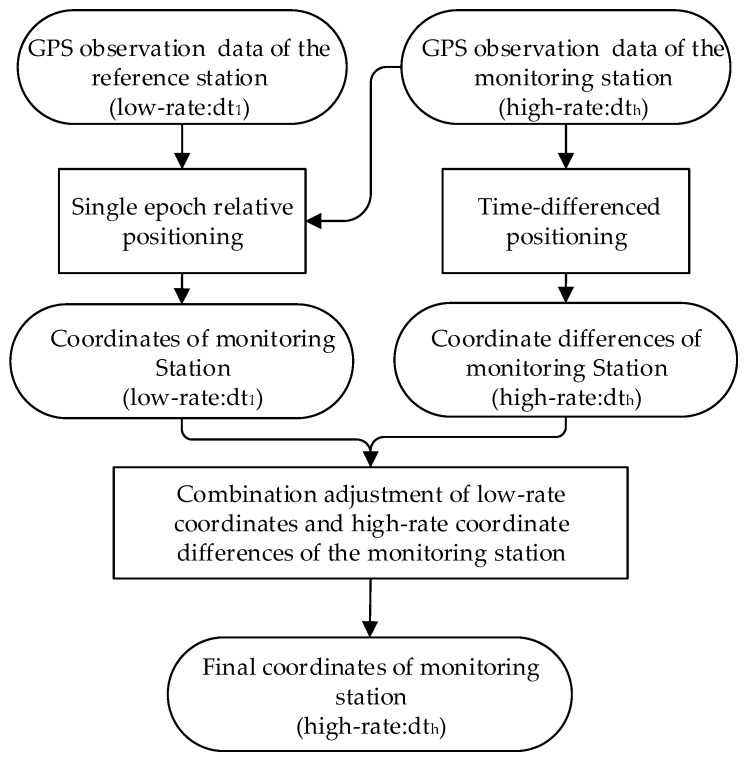
Data processing of combining high- and low-rate GPS position.

There are three steps in the data processing when combining high- and low-rate GPS receivers. Firstly, the low-rate position of the monitoring station is obtained by the traditional single-epoch relative positioning. Then, the monitoring station’s high-rate coordinate changes between sequential epochs are calculated by time-differenced positioning. Finally, we obtain the high-precision and high-rate coordinate time series of the monitoring station through combining the acquired low-rate coordinates and high-rate coordinate differences. The coordinate time series of the monitoring station subtracted by its reference coordinate value offers the response of tall buildings to the wind load. The entire data processing process is shown in Figure 2.

### 2.1. Single-Epoch Relative Positioning

In single-epoch relative positioning, carrier phase ambiguity resolution is the prerequisite for a high-precision position solution. Currently, there are mainly two ambiguity resolution methods, one is on-the-fly (OTF) method [24,25,26], and the other is single-epoch algorithm [27,28,29]. The OTF algorithm is generally based on multiple epoch observations. During this observation period, no cycleslips occur, or the cycleslips have been detected and repaired successfully. In addition, after successful ambiguity resolution, if the satellite signal is blocked or not locked, the ambiguity resolution will be reinitialized. The single-epoch algorithm generally sets up a small ambiguity search space with the help of a prior information. Based on the characteristics of wind load deformation of tall buildings, a single-epoch ambiguity resolution algorithm based on the combination of deformation characteristics and maximum deformation constraints is adopted in this paper [11]. The algorithm first determines the accuracy of the monitoring station position according to the maximum deformation characteristic and establishes the search ambiguity space Ω_1_. Then, based on the maximum deformation, Cholesky decomposition method is used to set up the ambiguity search space Ω_2_. Finally, the intersection of Ω_1_ and Ω_2_ is used as the ambiguity search space Ω. The algorithm can considerably reduce the ambiguity search space and effectively improve the ambiguity resolution success rate. The efficiency of the single-epoch algorithm will be improved obviously when the horizontal or vertical deformations are constrained [10].

### 2.2. Time-Differenced Positioning

Assuming that the monitoring station has n common GPS satellites from epoch i to i−1, the ionosphere-free phase observation vectors are $\Phi_{i}$ and $\Phi_{i-1}$, respectively; the vector of the differenced observation between the epochs is $\Delta \Phi_{i,i-1}=\Phi_{i}-\Phi_{i-1}$; $V_{i,i-1}$ is the residual vector of the time-differenced observation $\Delta \Phi_{i,i-1}$; $x_{i}$ and $x_{i-1}$ are unknown parameters corresponding to epoch *i* and *i* − 1, respectively, including three coordinate components of receiver and one receiver clock error parameters; $x_{i,0}$ and $x_{i-1,0}$ are the approximate values of $x_{i}$ and $x_{i-1}$, respectively; the correction vector of the unknown parameter are $dx_{i}=x_{i}-x_{i,0}$ and $dx_{i-1}=x_{i-1}-x_{i-1,0}$, respectively. Since the monitoring station uses high-rate GPS receivers, the observation time interval is very short. Neglecting the multipath effect and the residual tropospheric delay between epoch *i* and *i* − 1, the error equation for the time-differenced observation between the epochs can be expressed as

(1) $$V_{i,i-1}=A_{i}\cdot dx_{i}-A_{i-1}\cdot dx_{i-1}-\omega_{i,i-1}$$

where $A_{i}$ an **d**$A_{i-1}$ are the design matrices of epoch *i* and *i* − 1, respectively; $\omega_{i,i-1}$ is the observed-computed vector. Let $d\Delta x_{i,i-1}$ the difference between $dx_{i}$ and $dx_{i-1}$, their relationship can be described as

(2) $$dx_{i}=dx_{i-1}+d\Delta x_{i,i-1}$$

Inserting Equation (1) one can get

(3) $$V_{i,i-1}=A_{i}\cdot d\Delta x_{i,i-1}+\delta A_{i,i-1}\cdot dx_{i-1}-\omega_{i,i-1}$$

where $\delta A_{i,i-1}$ is the design matrix change between epoch *i* and *i* − 1 ($\delta A_{i,i-1}=A_{i}-A_{i-1}$). Due to the high altitude of GPS satellite orbits, the design matrix changes very slowly in a short period, and as a result the elements in $\delta A_{i,i-1}$ are very small. $dx_{i-1}$ is the correction value of the approximate monitoring station coordinate at epoch *i* − 1, since the approximate coordinates of the monitoring station at low rate can be determined by single-epoch relative positioning with centimeter precision, $dx_{i-1}$ will be in the range of centimeters to decimeters if reference station coordinates are accurate. Thus $\delta A_{i,i-1}\cdot dx_{i-1}$ is safely neglected. Then Equation (1) can be simplified as:

(4) $$V_{i}=A_{i}\cdot d\Delta x_{i,i-1}-\omega_{i}$$

Equation (4) is the function model for estimating the coordinate difference between adjacent epochs. After the function model is determined, a reasonable stochastic model of observations needs to be selected in the least squares data processing. In this paper, a stochastic model based on satellite elevation angle is selected. Assuming that the variance of the non-differenced ionosphere-free combination phase observation value in the zenith is $\delta_{0}^{2}$, then the variance of the phase observation at an elevation angle e is $\delta^{2}(e)$.

(5) $$\delta^{2}(e)=\frac{\delta_{0}^{2}}{\sin^{2}(e)}$$

Once high precision $d\Delta x_{i,i-1}$ is obtained, the coordinate difference $dx_{i,i-1}$ can be determined as

(6) $$dx_{i,i-1}=d\Delta x_{i,i-1}+(x_{i,0}-x_{i-1,0})$$

### 2.3. Combination Adjustment

It is assumed that sampling rate (low-rate) of the reference station is $dt_{l}$ in the kinematic relative positioning (5 s, 15 s, etc.), and the sampling rate (high-rate) of the monitoring station is $dt_{h}$(0.1 s, 1 s, etc.). Then m times of time-differenced positioning need to be performed between two adjacent low-rate observations in the monitoring station m is calculated by

(7) $$m=dt_{l}/dt_{h}$$

Assuming that the two adjacent observation epochs in low-rate are $t_{i}$ and $t_{i-1}$, the corresponding monitoring station coordinates processed by kinematic relative positioning are $x_{i}$ and $x_{i-1}$, respectively. During $t_{i}$ to $t_{i-1}$, there are m coordinate differences, $dx_{i-1|k}(k=1,m)$. Theoretically, the following Equation (8) should be satisfied between $x_{i}$, $x_{i-1}$, and $dx_{i-1|k}(k=1,m)$.

(8) $$x_{i-1}+\sum_{k=1}^{m}dx_{i-1|k}=x_{i}$$

As error exists in the positioning, Equation (8) cannot be met exactly, and there is a misclosure ε as follows.

(9) $$\varepsilon =x_{i}-(x_{i-1}+\sum_{k=1}^{m}dx_{i-1|k})$$

Considering the precision of $dx_{i-1|k}(k=1,m)$ obtained by the least squares method in a short period is constant, then misclosure ε can be evenly allocated to each $dx_{i-1|k}$. The coordinate difference between the adjacent epochs after misclosure correction ${\hat{dx}}_{i-1|k}$ is:

(10) $${\hat{dx}}_{i-1|k}=dx_{i-1|k}-\varepsilon /m$$

With low-rate monitoring station coordinates $x_{i}$, $x_{i-1}$ at epoch $t_{i}$ and $t_{i-1}$ and high-rate misclosure corrected monitoring station coordinate difference ${\hat{dx}}_{i-1|k}$ between epoch $t_{i}$ and $t_{i-1}$, high-rate monitoring station coordinate $x_{i-1|k}$ between epoch $t_{i}$ and $t_{i-1}$ can be computed by

(11) $$x_{i-1|k}=\sum_{n=1}^{k}{\hat{dx}}_{i-1|n}+x_{i-1}$$

Combining low-rate kinematic relative positioning results $x_{i-1}$ and high-rate time-differenced positioning results ${\hat{dx}}_{i-1|k}$, we can get high-rate kinematic positioning coordinates of the monitoring station between epoch $\;t_{i}$ and $t_{i-1}$.

We developed an in-house software for a simulation experiment (Section 3) and a real data experiment (Section 4). The detailed data processing strategies for GPS single-epoch relative positioning and time-differenced positioning are listed in Table 1.

## 3. Simulation Experiment

### 3.1. Experiment Design

**Figure 3:**
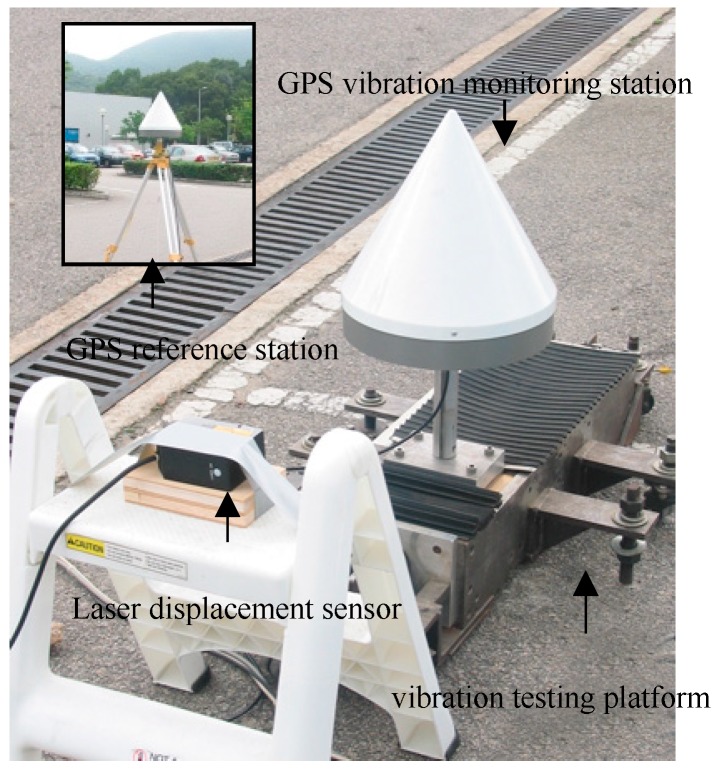
Setup of GPS simulation shaking experiment system.

The experiment is based on a vibration platform with two high-rate TOPCON GB-1000 dual frequency GPS receivers. One is installed in the vibration platform as a monitoring station, and the other is set as a reference station about ten meters away. Both the reference and monitoring station are located on the same open ground. The CR-3 Chock Ring Antenna is used to reduce multipath effect, and data sampling rate is 10 Hz. In order to compare the results, the laser ranging sensor is used to measure vibration displacement of the vibration platform directly. Figure 3 shows the GPS simulated shaking experiment system. Under motor control, the vibration platform has sinusoidal vibrations along the north direction with step-wise varying frequencies and amplitudes, simulating the wind-induced vibrations of the tall building. Due to restricted space, this paper only selects datasets with vibration frequency of 0.3 Hz for analysis. 

### 3.2. Data Processing and Result Analysis 

GPS positioning provides Cartesian coordinates (*X*, *Y*, *Z*). In order to facilitate the comparison and analysis with the laser ranging sensor, Cartesian coordinates (*X*, *Y*, *Z*) can be transformed to topocentric coordinate system ENU (east, north, up) by Equation (12). In this experiment, coordinates change in *N* direction represents the vibration displacement of the shaking table.

(12) $$\left[\begin{array}{c} E\\ N\\ U \end{array}\right]=M\left[\begin{array}{c} X-X_{0}\\ Y-Y_{0}\\ Z-Z_{0} \end{array}\right]$$

The coordinate transformation matrix *M* in Equation (12) is

(13) $$M=\left[\begin{array}{ccc} -\sin\lambda_{0} & \cos\lambda_{0} & 0\\ -\sin\varphi_{0}\cos\lambda_{0} & -\sin\varphi_{0}\sin\lambda_{0} & \cos\varphi_{0}\\ \cos\varphi_{0}\cos\lambda_{0} & \cos\varphi_{0}\sin\lambda_{0} & \sin\varphi_{0} \end{array}\right]$$

where $\left(X_{0},Y_{0},Z_{0}\right)$ is the Cartesian coordinates of the origin of the ENU coordinate system. $\lambda_{0}$ and $\varphi_{0}$ are WGS84 geodetic longitude and latitude respectively. In order to evaluate the GPS monitoring methods mentioned above, we processed the experimental data in the following three schemes.

- Scheme 1: 10 Hz observation data of reference and monitoring stations are processed by single-epoch relative positioning technology (hereafter referred to as relative positioning method).
- Scheme 2: 10 Hz observation data of monitoring station is processed by the PPP technology (hereafter referred to as the PPP method).
- Scheme 3: 30 s observation data of the reference station and the 10 Hz observation data of the monitoring station are processed by the combining high- and low-rate GPS positioning method proposed in this paper (hereafter referred to as the combining high- and low-rate GPS method).

**Figure 4:**
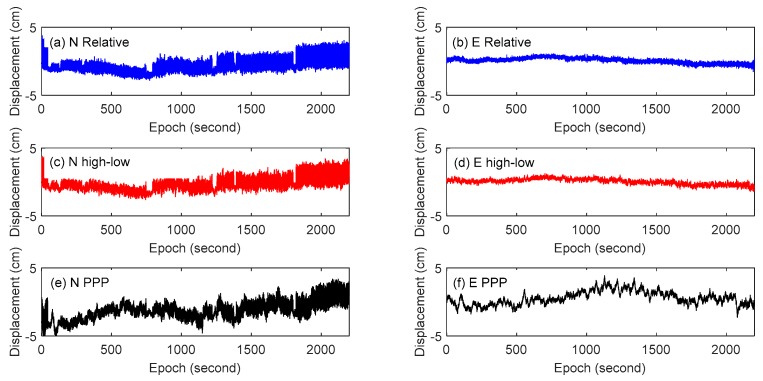
Displacement time series of GPS: (**a**)Relative positioning method in the N direction, (**b**) relative positioning method in the E direction, (**c**) combining high- and low-rate GPS method in the N direction, (**d**) combining high- and low-rate GPS method in the N direction, and (**e**) PPP method in the N direction, (**f**) PPP method in the E direction.

The displacement time series of the three schemes in the N and E directions are shown in Figure 4. In the N and E directions, the result of combining high- and low-rate GPS method is highly similar to the result of the relative positioning method, while the result of the PPP method is quite different from the former two methods. In fact, monitoring station on vibration table has no displacement in the east direction, but the result of the PPP method has obvious fluctuation. It is verified that PPP method has obvious defects in monitoring quasi-static deformation. The results of the combining high- and low-rate GPS method in the east direction show that the proposed method performs similarly compared with the traditional single-epoch relative positioning method in monitoring the quasi-static deformation.

**Figure 5:**
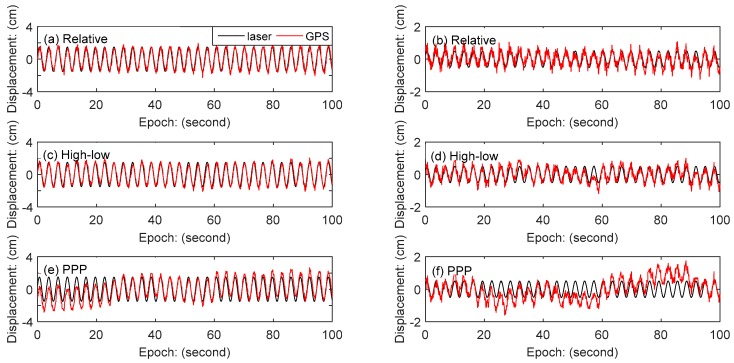
Comparison of N-direction displacement observed by GPS and laser sensor, with the results of the amplitude 15 mm on the left and the results of the amplitude 5 mm on the right: (**a**,**b**) relative positioning method, (**c**,**d**) combining high- and low-rate GPS method, (**e**,**f**) PPP method (note the y-axis scales are different).

There are 4 step-wise vibrations with amplitudes of 5 mm, 10 mm, 15 mm and 20 mm in the north direction. For brevity, we only chose 2 representative vibration amplitudes (15 mm and 5 mm) for analysis. The local amplification of the vibration series with amplitudes of 15 mm and 5 mm in the north directionis compared with the monitoring displacement of laser sensor, as shown in Figure 5. The result shows that the greater the vibration displacement is, the better the result of GPS coincides with the result of laser displacement sensor. In the three GPS data processing schemes, the result of the relative positioning method is highly consistent with that of the mixed method. Even if the amplitude is 5 mm, results of the two methods have good agreement with laser displacement sensor in vibration displacement monitoring. The PPP method has the worst performance in the three processing schemes, especially when the amplitude is 5 mm, the result has obvious deviation with results of the laser displacement sensor.

**Figure 6:**
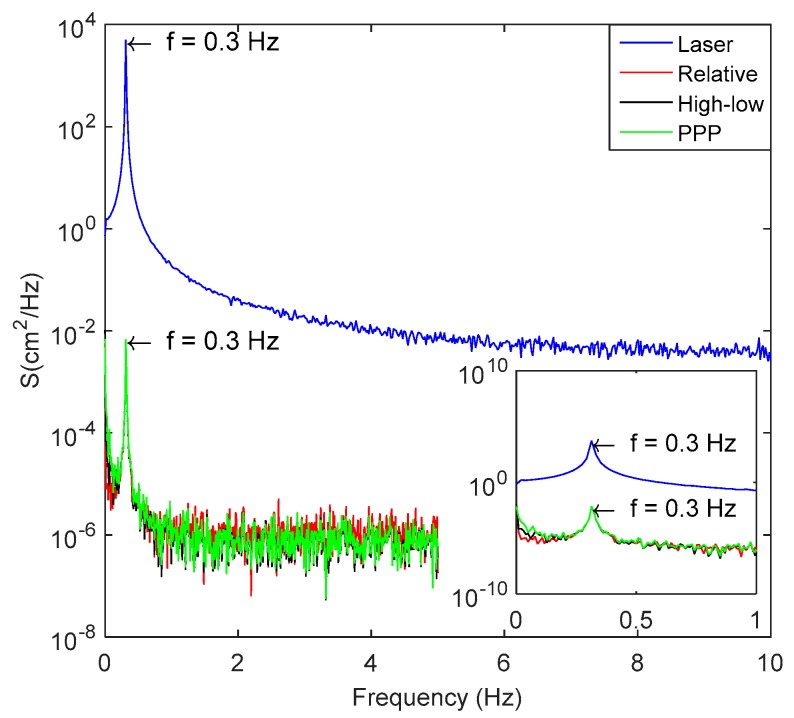
Spectrum analysis of N-direction vibration displacement series.

Based on the fast Fourier transform (FFT) method, the spectrum of the GPS displacement time series in the north direction is provided in Figure 4 and the observation of the laser ranging sensor is also analyzed. The results are shown in Figure 6. As can be seen, all the three methods can accurately detect the vibration frequency of 0.3 Hz and have a good consistency with the result of the laser sensor. However, in the low frequency part, the GPS results show a higher energy density, especially the PPP positioning method, which is mainly caused by the residual error of GPS positioning, especially low frequency GPS multipath error.

## 4. Experiment with Real Data

### 4.1. Data Acquisition

**Figure 7:**
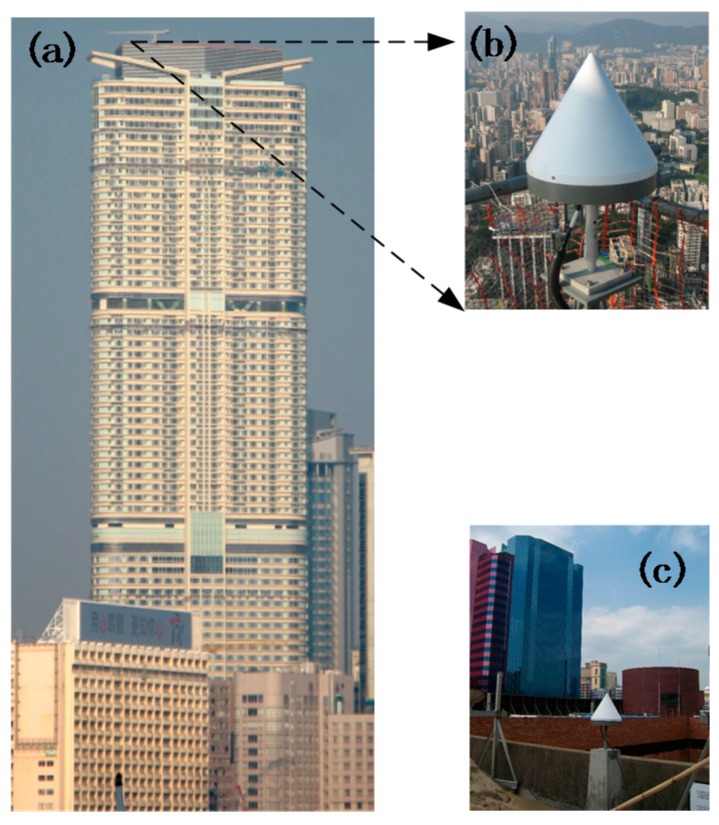
GPS monitoring system: (**a**) a monitored tall building; (**b**) GPS monitoring station; and (**c**) GPS reference station.

**Figure 8:**
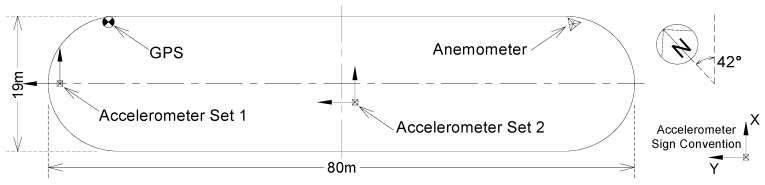
Plane view of test building and field measurement configuration.

The Hong Kong University of Science and Technology CLP Wind Tunnel Laboratory conducted a long-term wind-borne dynamic deformation monitoring of a high-rise building in Hong Kong. The building is rectangular-shaped and has 68 floors, with a height of about 260 m, and the ratio of length to width is 4:1. The surrounding environment is relatively open and the building is susceptible to wind loads. The X direction of the building is 42 degrees north west. The data acquisition instruments installed in the entire monitoring system include two TOPCON GB-1000 dual-frequency GPS receivers, four Honeywell QA-650 accelerometers, and a GILL supersonic anemometer (unfortunately the anemometer did not work properly during the experiment). To monitor the wind-induced dynamic deformation of the building, two GPS receivers were used to form a baseline. One was installed on the roof of the building as a monitoring station, and the other was installed at the Hong Kong Polytechnic University as a reference station. The baseline length is approximately 0.84 km. The GPS monitoring system is shown in Figure 7. Four accelerometers were used to monitor the acceleration of the wind-induced vibration of the building. They were installed in two groups on the wing and center of the top floor of the building. The anemometer was installed at the highest point on the roof of the building to observe the wind speed and direction. The GPS sampling rate was set to 10 Hz, and the accelerometer and the anemometer were set to 20 Hz. The entire system was an automatic continuous monitoring system. The layout of the instrument is shown in Figure 8.

### 4.2. Analysis of Monitoring Results

We selected one hour of experiment data from10:00 to 11:00 (local time) on 6 August 2008 to perform analysis. It was cloudy and the temperature was about 25–27 °C. A tropical storm happened in the day. At that time, this building was not in use and was less affected by surrounding traffic. It is considered that the building deformation during the experiment is mainly caused by the wind. In order to verify the superiority of our proposed method in measuring the wind-induced response of tall buildings, we adopt three GPS data processing schemes in the above simulation test. This paper analyzes the results in the three aspects: identifying the modal parameters of tall buildings, observation of building vibration displacement, and the extraction of quasi-static deformation. In order to facilitate the comparison with the data observed by the accelerometer, the coordinates obtained by GPS solution were converted from WGS-84 coordinates to the building coordinate system established in Figure 8.

#### 4.2.1. Modal Parameter Identification

**Figure 9:**
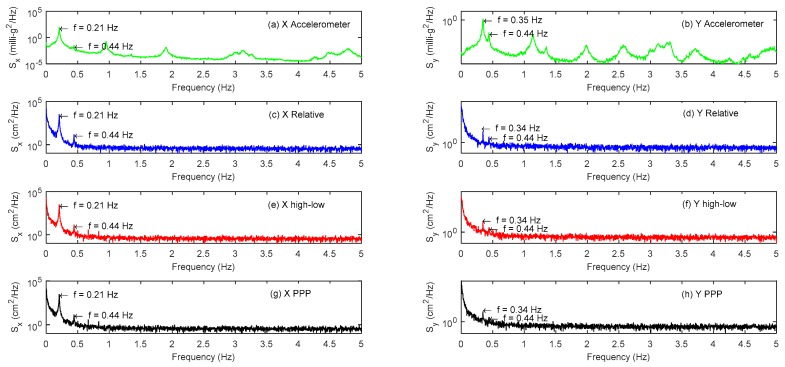
Spectrum analysis of GPS and accelerometer observation sequence: (**a**) X direction acceleration data; (**b**) Y direction acceleration data; (**c**) X direction relative positioning method; (**d**) Y direction relative positioning method; (**e**) X direction combining high- and low-rate GPS method; (**f**) Y direction combining high- and low-rate GPS method; (**g**) X direction PPP method; and (**h**) Y direction PPP method.

The vibration frequencies of the first three main modals of high-rise buildings under external excitation conditions are very important for their design and operation safety. The one hour of acceleration data and its corresponding GPS displacement results are analyzed by Fast Fourier Transform (FFT), and the results are shown in Figure 9. Figure 9a,b shows the results of two groups of accelerometers and Figure 9c,d shows the results of relative positioning method. Figure 9e,f shows the results of combining high- and low-rate GPS method, and Figure 9g,h shows the results of PPP method. As the accelerometer is very sensitive to vibration and is less disturbed by the external noise, it has an advantage over GPS in building modal identification. This paper takes accelerometer results as a reference. From subgraphs (a) and (b), it can be concluded that the vibration frequencies of the first three main modals of the tall building are 0.21 Hz, 0.35 Hz, and 0.44 Hz respectively. Comparing the GPS with its accelerometer results, it can be seen that all three GPS data processing schemes can accurately identify the main modal parameters of the monitored building, and because GPS is greatly affected by residual errors, it exhibits a higher energy spectral density in low frequency and the vibration information cannot be recognized in high frequency. The GPS spectrum results of the three methods are similar, but the results of combining a high- and low-rate GPS method is relatively closer to the results of relative positioning method and have a clearer main modal frequency than the PPP method, especially in the third modal.

#### 4.2.2. Wind-Induced Vibration Displacement Analysis

**Figure 10:**
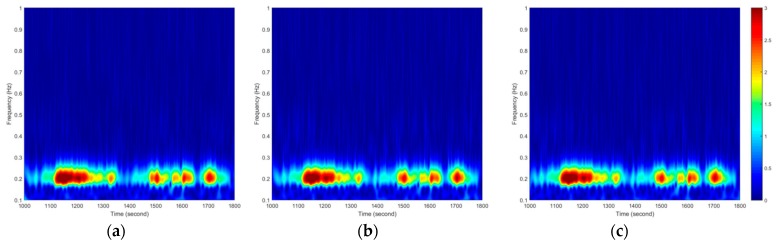
Spectrum of GPS displacement time history (0~1 Hz band) in the X direction: (**a**) relative positioning method, (**b**) combining high- and low-rate GPS method, and (**c**) PPP method.

According to the above analysis, the main modal frequency parameters of this high-rise building are less than 1 Hz, so the cut-off frequency is set to 1 Hz in Figure 10. Figure 10 shows that three GPS data processing methods can clearly observe the main modal frequency in the X direction (about 0.2 Hz), and the energy spectral densities of the three GPS methods are highly consistent with time.

**Figure 11:**
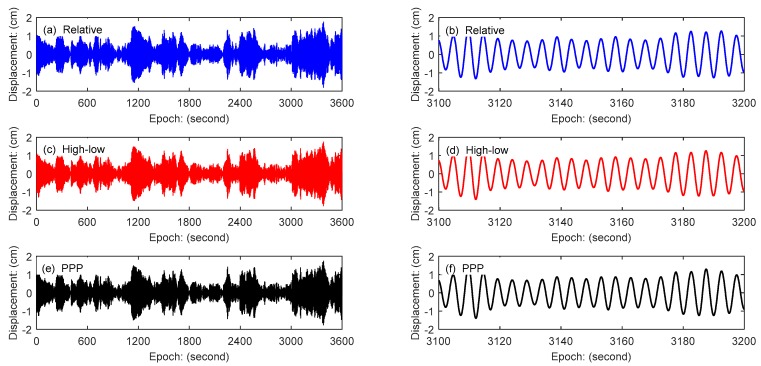
Displacement comparison of the first modal in the X direction: (**a**) relative positioning method, (**b**) is enlargement of the detail of (**a**); (**c**)combining high- and low-rate GPS method, (**d**) is enlargement of the detail of (**c**); (**e**) PPP method, and (**f**) is enlargement of the detail of (**e**).

In order to obtain the first modal wind-induced vibration displacement of the building, the sym25 (sym 2 by 5) wavelet packet of MATLAB (MathWorks, Natick, MA, USA) is used to decompose the GPS data into 5 levels, and the data in the frequency band (0.15625~0.3125 Hz) is extracted. The results are shown in Figure 11, in which the right-side illustration is an enlargement of the detail of the left result. According to Figure 11, it can be seen that the three GPS data processing schemes show a high consistency in numerical comparisons of the whole or the details. Therefore, the combining high- and low-rate GPS technology proposed in this paper can reliably monitor the wind-induced vibration displacement of high-rise buildings.

**Figure 12:**
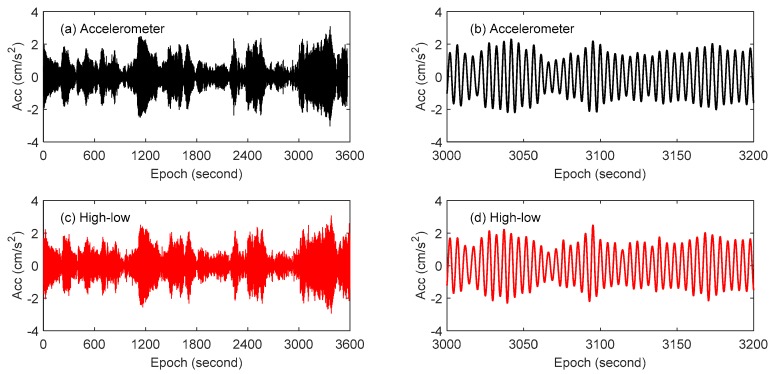
Acceleration comparison of GPS and Accelerometer: (**a**) accelerometer result, (**b**) is enlargement of the detail of (**a**); (**c**) combining high- and low-rate GPS method, (**d**) is enlargement of the detail of (**c**).

The accelerometer is sensitive to vibration and its observation precision is high. In order to compare the displacement data measured by GPS with the accelerometer data, the displacement data in Figure 11 was derived twice. The sym25 wavelet packet of MATLAB (MathWorks, Natick, MA, USA) is also used to decompose the acceleration data, and the data in the same frequency band of GPS was extracted. The comparison of the acceleration of the first modal obtained by combining high- and low-rate GPS technology with the acceleration observed by the accelerometer is shown in Figure 12. The results show that these two methods are highly consistent. Comparing with acceleration data, the accuracy and reliability of the proposed method applied to wind-induced vibration monitoring of tall buildings are further verified.

#### 4.2.3. Wind-Induced Quasi-Static Deformation Analysis 

**Figure 13:**
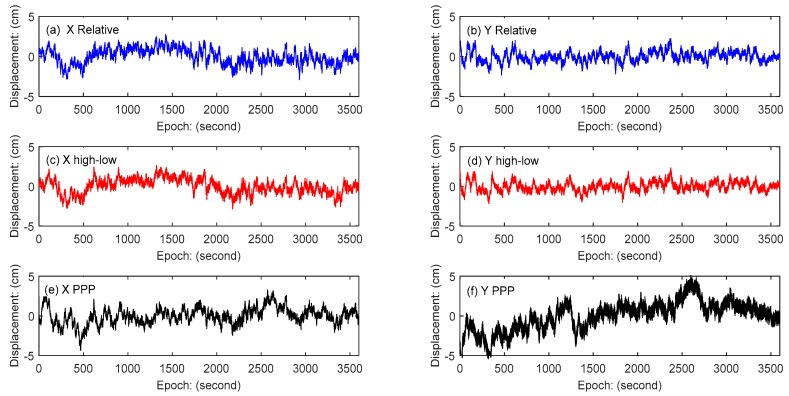
Quasi-static displacement time series: (**a**) X direction relative positioning method, (**b**) Y direction relative positioning method; (**c**) X direction combining high- and low-rate GPS method, (**d**) Y direction combining high- and low-rate GPS method; (**e**) X direction PPP method, (**f**) Y direction PPP method.

The wind-induced response of tall buildings includes the buildings’ vibration of the natural modal and the quasi-static deformation. From the above analysis, it can be concluded that these three GPS data processing methods can accurately observe the vibration part displacement of the building. The quasi-static deformation information can be obtained by subtracting the vibration part from the total monitored displacement, as shown in Figure 13. The results show that the relative positioning method and the combining high- and low-rate GPS positioning method have good consistency in the quasi-static deformation displacement monitoring both in the X direction and the Y direction. Compared with the other two methods, the PPP method has a larger fluctuation in the coordinate displacement time series, especially in the Y direction of the building, which is not obvious to the wind load. This is mainly caused by the residual error in PPP. The results also show that the building is more sensitive to the wind load in the X direction than the Y direction due to building shape, so the deformation in the X-direction has a systematic variation caused by wind load and the deformation in the Y-direction fluctuates randomly near the zero.

It should be pointed out that multipath is the main obstacle to get accurate quasi-static deformation [30]. In order to mitigate multipath effect, firstly, we select the monitoring and reference station carefully for the experiment. Secondly, we use moving average technique to get the quasi-static deformation from high-rate displacement. As such, multipath effect will be reduced to some extent in our experiments. To further improve quasi-static deformation accuracy, multipath should be considered in our successivestudy.

## 5. Conclusions

Precisely monitoring the dynamic response of tall buildings under wind load can not only assess their health in real time, but also provide valuable reference for relevant wind resistance design. GPS technology offers three-dimensional displacements of the monitored object and has many advantages over the traditional monitoring methods. This paper proposes a new monitoring method of wind-induced response of tall buildings by combining high- and low-rate GPS receivers. The method uses the existing urban CORS stations with low sampling rate as the reference station. Low-rate coordinates of the monitoring station are obtained by using GPS single-epoch positioning technology, and the high-rate coordinate changes of the monitoring station are generated by time-differenced positioning. The coordinates of different rates derived are finally integrated to provide high-rate and high-accuracy position time series. Simulation experiment and real building wind-induced vibration monitoring were carried out. Results show that the new method and existing methods have a good agreement in the vibration frequency identification, displacement, and acceleration. The quasi-static deformation measured by the new method during typhoon is consistent with GPS relative positioning approach, and the precision is higher than the PPP method. The proposed method is easy to realize and can reduce the monitoring costs, with a comparable positioning accuracy to the traditional single-epoch relative positioning method.

## Figures and Tables

**Table 1:** Data processing strategies.

Item	Single-Epoch Relative Positioning	Time-Differenced Positioning
Observations	Double-differenced L1 and L2 code and phase	Time-differenced ionosphere-free phase combination
Observation weight	Elevation dependent weight	Elevation dependent weight
Satellite obit and clock	Broadcast ephemeris	Precise orbit and clock from IGS
Tropospheric delay	Saastamoinen model	Saastamoinen model
Mapping function	Global Mapping Function (GMF)	Global Mapping Function (GMF)
Ionospheric delay	Neglected	First order effect eliminated by ionosphere-free linear combination
